# Efficacy and safety of Guben Tongluo formula in treating chronic kidney disease (stage G3): a multicenter randomized controlled clinical trial

**DOI:** 10.3389/fmed.2025.1691637

**Published:** 2025-10-24

**Authors:** Lianxiang Duan, Ziyang Liu, Ling Chen, Yiqing Yang, Yenan Fan, Jie Chen, Jing Hu, Xuezhong Gong, Yue Guo

**Affiliations:** ^1^Department of Nephropathy, Seventh People’s Hospital of Shanghai University of Traditional Chinese Medicine, Shanghai, China; ^2^Department of Nephrology, Shanghai Municipal Hospital of Traditional Chinese Medicine, Shanghai University of Traditional Chinese Medicine, Shanghai, China

**Keywords:** efficacy and safety, Guben Tongluo formula, chronic kidney disease, multicenter randomized controlled clinical trial, traditional Chinese medicine

## Abstract

**Background:**

The incidence of chronic kidney disease (CKD) is increasing yearly; however, an effective drug treatment method for early-stage CKD (stage G3) is lacking. Thus, we aimed to determine the efficacy and safety of Guben Tongluo formula (GTF) for patients with CKD (stage G3).

**Methods:**

One hundred and twenty participants were enrolled and randomly divided into losartan potassium (LP) group and LP + GTF group. LP group received general treatments combined with LP, and LP + GTF group received general treatments combined with GTF and LP. Evaluation indicators included changes in renal function, serum lipid, inflammatory factors, oxidative damage, renal fibrosis, traditional Chinese medicine (TCM) symptom scores, and clinical effective rate. In addition, vital sign indicators and adverse events (AEs) were closely observed throughout the study.

**Results:**

Scr and BUN levels were significantly lower and eGFR were significantly improved in LP + GTF group (*p* < 0.05). There was no statistically significant difference of UA and 24 h U-pro levels (*p* > 0.05). TG, TC levels were significantly lower and HDL-C levels were significantly higher in LP + GTF group (*p* < 0.01). No statistically significant difference in LDL-C levels was observed (*p* > 0.05). Inflammatory factors levels were significantly lower in LP + GTF group (*p* < 0.01). Notable increases in HO-1 and SOD levels were observed in LP + GTF group (*p* < 0.01), and MDA levels showed no statistically significant difference (*p* > 0.05). CTGF and TGF-β1levels were significantly lower in LP + GTF group (*p* < 0.01), while no significant difference was observed of PC-III and Col-IV levels (*p* > 0.05). TCM syndrome scores in LP + GTF group were significantly lower (*p* < 0.01). The clinical efficiency rate in LP + GTF group (73.3%) was better than that in LP group (40%). No significant between-group differences were observed in AEs.

**Conclusion:**

LP + GTF group demonstrated a better clinical efficacy rate than LP group. GTF regulates serum lipid levels and has anti-inflammatory, antioxidative, and anti-renal fibrosis effects. GTF showed potential benefits in this small multicenter randomized controlled trial, warranting confirmation in larger, fully blinded randomized trials.

## 1 Introduction

Chronic kidney disease (CKD) is a condition characterized by impaired kidney structure and function, triggered by various etiological factors that may ultimately progress to end-stage renal disease (ESRD) (1, 2). In 2022, the global prevalence of CKD was estimated at ~850 million people (1). In the advanced stages of CKD, patients rely solely on kidney replacement therapy (KRT) to sustain their lives (3, 4). However, the widespread adoption of KRT in China remains hindered by factors such as cost, technological limitations, and trauma, and life-threatening complications remain prevalent even for patients receiving KRT (5–7). CKD has thus imposed a substantial social and economic burden and has emerged as a critical global health threat (8–12).

CKD stages are defined by varying levels of the estimated glomerular filtration rate (eGFR), reflecting the degree of renal function deterioration (13–15). In early CKD stages (stage G3), eGFR declines modestly, and proteinuria serves as an independent risk factor for disease progression, often accompanied by hematuria (16, 17). The hallmark pathological features of CKD include extracellular matrix accumulation and renal fibrosis, with renal function progressively deteriorating (18–21). Renal fibrosis underpins the progression of CKD, with inflammation and immune responses contributing to its exacerbation (22–24). Thus, mitigating renal fibrosis is central to improving patient outcome. Current Western medical approaches to managing early-stage CKD involve comprehensive interventions, utilizing ACE inhibitors (ACEI) or angiotensin receptor blockers (ARB) to reduce proteinuria, along with efforts to control blood glucose, blood pressure, lipids, and malnutrition (25–28). Recent clinical and experimental studies have demonstrated that traditional Chinese medicine (TCM) effectively delays CKD progression, offering distinct advantages as a promising therapy (29–32). TCM is more versatile in treating CKD, exhibiting fewer side effects, and achieving efficacy through the coordination of multiple mechanisms and targets (33–36).

Guben Tongluo formula (GTF) is an empirical formulation developed by Professor Liqun He, a renowned TCM practitioner from Shanghai (37, 38). GTF works by tonifying the spleen and kidney, removing blood stasis, and expelling turbidity, and has been particularly effective in treating early-stage CKD with proteinuria as a primary symptom. A clinical study demonstrated that GTF significantly alleviates hematuria and proteinuria while improving renal function in patients with IgA nephropathy, likely through the modulation of inflammation and immune responses (39). Previous *in vivo* studies suggest that reno-protective effects of GTF, including the reduction of urinary protein excretion, may be attributed to its ability to protect podocytes and downregulate NLRP3 inflammatory factors (40). Losartan potassium (LP), the first angiotensin-2 receptor antagonist used for hypertension treatment, alleviates vascular tension, reduces peripheral resistance, and lowers blood pressure. Additionally, LP significantly reduces the combined cardiovascular risk in hypertensive patients and offers certain reno-protective effects, potentially lowering the risk of ESRD (41). This study aimed to assess the efficacy and safety of GTF in treating stage G3 CKD through a multicenter randomized controlled trial and to explore the underlying therapeutic mechanisms of GTF.

## 2 Methods

### 2.1 Study design and setting

**Figure 1:**
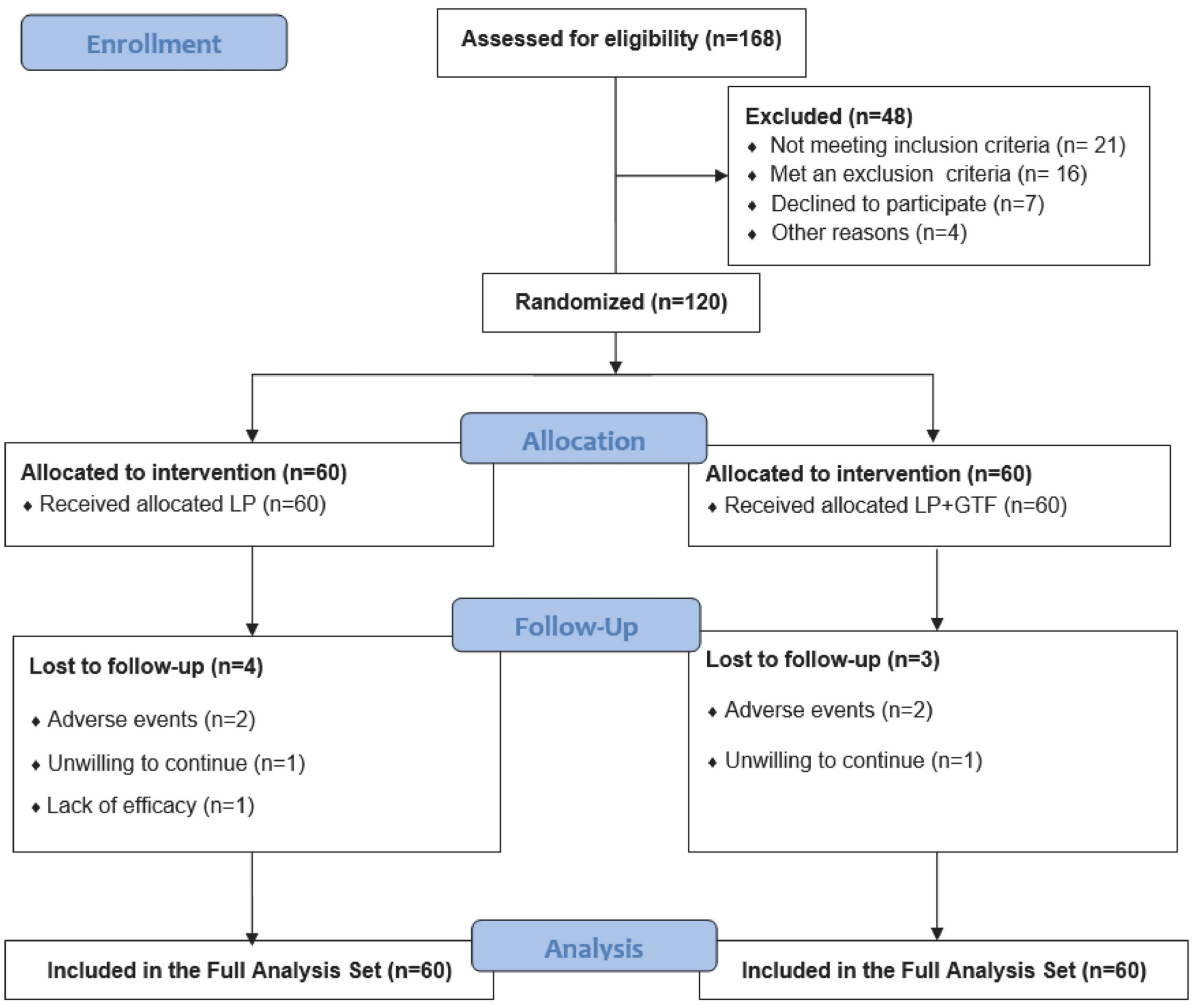
Flow chart of the study.

This was a multicenter randomized controlled clinical trial, and the study protocol was approved by the ethics committee of each participating hospital with the Ethics Committee of Seventh People’s Hospital of Shanghai University of Traditional Chinese Medicine (No. 2023-7th-HIRB-008). This study was conducted in accordance with the Declaration of Helsinki (42) and relevant Chinese regulations on clinical trial research, respecting the rights of patients and requiring the completion of patients’ informed consents. It has been registered in the Chinese Clinical Trial Registry (no. ChiCTR2400090125). The flowchart of the study is shown in Figure 1.

### 2.2 Participants

The study duration ranged from 15 December 2023 to 30 May 2025. Participants were recruited from the Department of Nephrology of each hospital. The three hospitals were as follows: (1) Seventh People’s Hospital of Shanghai University of Traditional Chinese Medicine, (2) Shanghai Pudong New Area Hudong Community Health Service Center, and (3) Shanghai Pudong New Area Jin Yang Community Health Service Center. The patients were from three hospitals above and were diagnosed with CKD (stage G3), with a total of 120 cases. CKD patients [30 ≤ eGFR<60 mL/(min·1.73m^2^)] with spleen-kidney qi deficiency and blood stasis syndrome was collected and divided into 2 groups: LP group and LP + GTF group, with 60 cases in each group. To guarantee the target sample size, recruitment posters will be posted and trial advertisements will be accessible through a certain web page. Before participating in the trial, a specialized clinical researcher will inform participants of the inclusion and exclusion criteria, relevant treatments and examinations to be completed, as well as the potential benefits and risks. In addition, the clinician will make every effort to prevent the harm that might be caused to participants by this study, and ensure that every participant has signed the informed consent. The documents of the participants will be coded and personal information will not be disclosed.

#### 2.2.1 Diagnostic and staging criteria for CKD

According to the diagnostic criteria of the *Kidney Disease: Improving Global Outcomes (KDIGO)* CKD Work Group (43), CKD is defined as one of the following conditions that has persisted for >3 months: (a) kidney damage, including albuminuria (urinary albumin excretion rate≥30 mg/24 h; urinary albumin creatinine ratio≥3 mg/mmol), abnormal urinary sediment, renal tubule related lesions, abnormal histological examination, abnormal imaging examination, kidney transplantation history; (b) eGFR decreased: 30 mL/(min·1.73m^2^) ≤ eGFR<60 mL/(min·1.73m^2^). CKD staging is recommended according to the KDIGO staging criteria (44).

#### 2.2.2 Diagnostic criteria for TCM syndrome differentiation

The TCM diagnostic criteria for CKD individuals with spleen-kidney qi deficiency and blood stasis syndrome are based on the guidelines provided in “*Diagnosis, Dialectical Classification, and Efficacy Evaluation of Chronic Renal Failure*” (45), which are outlined as follows:

(1) Primary symptoms: fatigue, weakness in the lower back and knees, persistent edema, abdominal distension. Secondary symptoms: clear nocturnal urine, sensitivity to cold and heat, loose stools; Tongue: pale or purplish; Pulse: fine and dull. (2) Concurrent blood stasis syndrome. Symptoms: dusky skin, dark purple lips and nails, fixed or aching back pain; Tongue: dark purple or petechiae or spots; Pulse: astringent or fine astringent pulse. To diagnose TCM syndrome, patients must present with either two primary symptoms or one primary symptom along with two secondary symptoms, one secondary symptom and one concurrent syndrome.

#### 2.2.3 Inclusion criteria

Patients were required to meet the following criteria for inclusion in this trial: (1) diagnosed with CKD (stage G3) based on Western medical diagnostic criteria and TCM syndrome differentiation; (2) aged 18–70 years, with generally good social compliance; (3) those who did not receive combined treatment with other TCM decoctions; (4) 24-h urinary protein excretion <2.0 g; and (5) signed informed consent, and voluntary participation.

#### 2.2.4 Exclusion criteria

The following conditions excluded patients from participation in the trial: (1) secondary CKD or patients with more severe multi-organ damage than primary CKD; (2) recent infections, or inability to control hypertension or hyperglycemia despite medication; (3) current severe renal anemia, acid–base imbalances, water-electrolyte disorders, or other major diseases. Patients with underlying conditions such as diabetes, hypertension, or hyperlipidemia that do not affect trial outcomes are not excluded; (4) circumstances that may complicate participation, such as changes in work environment or situations leading to difficulty in maintaining contact with the research team; (5) history of alcohol or drug dependence, or current abuse, identified through medical history. (6) sensitivity to multiple foods or drugs, or known serious allergies to ingredients in the study medication; (7) pregnant or breastfeeding women; and (8) participation in clinical trials of other drugs.

#### 2.2.5 Withdrawal criteria

Patients were withdrawn from the study under the following conditions: (1) failure to meet the diagnostic or inclusion criteria; (2) diagnosis of incomplete data that affected the assessment of efficacy and safety; (3) issues with patient compliance during the observation period; (4) initiation of treatment with other medications due to serious complications; and (5) discontinuation of treatment or the assigned drug due to adverse reactions.

To maintain the rigor and standardization of the research, the researchers provided a detailed explanation for each patient’s withdrawal and ensured proper archiving of patient report forms and other data for future review. Patients who were excluded from the final analysis were also excluded from the efficacy statistical analysis. However, for patients excluded owing to adverse reactions, a thorough investigation into the cause was conducted and documented after ensuring that all data had been accurately recorded.

#### 2.2.6 Shedding and trial suspension criteria

The following conditions led to shedding: (1) allergic reactions to the study drug, (2) natural attrition or loss to follow-up, and (3) occurrence of complications during the study, leading to automatic discontinuation of the clinical trial. In cases of shedding, the specific causes were carefully recorded in the research report. Data were divided according to the latest diagnostic indicators, followed by full statistical analysis. The trial was suspended under the following circumstances: (1) if a participant experienced a major adverse event (AE) that significantly impaired their normal quality of life, and (2) if a major procedural error occurred during the study that led to significant deviation from the research protocol or clinical practice.

Researchers were required to explain the reasons for each case of shedding and to ensure the preservation of relevant case report forms (CRFs) and other documentation for future reviews. All adverse reactions that occurred during the clinical study were recorded and analyzed meticulously. If a patient failed to return for a scheduled visit or lost contact, immediate attempts were made to reach the patient or their family members using alternative means (e.g., WeChat) to determine the cause of non-compliance.

### 2.3 Randomization and blinding

The randomization was performed by a designated research assistant utilizing a random number table generated through SPSS software (version 29.0; IBM Corp., Armonk, NY, USA). Participants were allocated at random in a 1:1 ratio to either the LP group or the LP + GTF group according to their order of admission. An independent statistician, who was not blinded, prepared the random allocation sequence. To ensure detailed allocation concealed, sealed envelopes containing sequential coding numbers were adopted. The unique properties of TCM decoctions made it impossible to blind the therapists and patients. To preserve the trial’s integrity, the outcome evaluators and data analysts were unaware of the treatment assignments. Both the participants and researchers were blinded to the group assignments throughout the study. When dispensing the drugs, the staff were unaware of the group assignments, ensuring blinding of both the grouping and dosing procedures. The intent-to-treat (ITT) population included all patients who were randomly assigned. The safety population consisted of all patients who had been administered any amount of the study drugs. In the full analysis set (FAS) population, demographics, baseline characteristics, and secondary efficacy endpoints were summarized. Safety outcomes were evaluated among the safety population, with no imputation for missing data.

### 2.4 Interventions

#### 2.4.1 Treatment methods

##### 2.4.1.1 General treatments

(1) Dietary management: a high-quality, low-protein diet (0.6 g/kg/d), low sodium intake (< 6 g/d), and caloric intake of 30–35 kCAVkg/d, ensuring nutritional requirements and a positive nitrogen balance without exacerbating azotemia. (2) Hypoglycemic therapy: Insulin, glipizide, or repaglinide should be utilized to effectively manage blood glucose, maintaining fasting blood glucose levels between 5.6 and 7.0 mmol/L, and glycosylated hemoglobin within the range of 4–6%. (3) Antihypertensive therapy: appropriate use of calcium channel blockers, α- or β-receptor antagonists, diuretics, and central antihypertensive agents to maintain blood pressure below 130/80 mmHg. (4) Symptomatic management: in accordance with guidelines, anemia and calcium and phosphorus metabolism disturbances should be appropriately addressed, and acid–base balance, along with water and electrolyte homeostasis, should be maintained to ensure normal blood lipid levels.

##### 2.4.1.2 LP group treatments

In addition to the general treatments, LP tablets (100 mg) were administered orally once daily.

##### 2.4.1.3 GTF + LP group treatments

In addition to the general treatments, GTF was administered orally in, one bag twice daily, while LP was administered as described above.

The manufacturer of LP tablets is Organon Pharma (UK) Limited (United Kingdom) and the batch number is Z006913. Formulation of GTF: raw Astragalus 15 g, Salviae Miltiorrhiza 15 g, Peach Kernel 10 g, Herba Lycopi 10 g, Glossy Privet Fruit 15 g, *Eclipta Alba* 15 g, Rhizoma Imperatae 30 g, *Rheum Officinale* 30 g, Ramuli Euonymi 15 g, among other ingredients. GTF was manufactured by Shanghai Wanshicheng Pharmaceutical Products Co., Ltd., with 200 mL/bag.

The treatment and follow-up lasted for 6 months. When participants are enrolled, all the indicators listed in the manuscript will be examined and recorded to ensure that the baseline data are balanced and comparable. At the third month, the safety indicators will be checked to ensure that no adverse reactions occur due to the administration of the drugs in the study. In the last week of the sixth month, all the indicators will be re-examined and compared with those before treatment to clearly determine the treatment effect.

### 2.5 Outcome measurements

#### 2.5.1 Evaluation outcome indicators

(1) Renal function related indicators: serum creatinine (Scr), blood urea nitrogen (BUN), serum uric acid (UA), serum albumin, eGFR; 24 h urine protein quantification (24 h U-pro); (2) serum lipid indicators: triglyceride (TG), cholesterol (TC), high-density lipoprotein cholesterol (HDL-C), and low-density lipoprotein cholesterol (LDL-C); (3) inflammatory factors: inflammatory cytokines including interleukin-1β (IL-1β), interleukin-6 (IL-6) and tumor necrosis factor-α (TNF-α); (4) oxidative damage indicators: hemeoxygenase-1 (HO-1), Superoxide dismutase (SOD) and malondialdehyde (MDA); (5) renal fibrosis indicators: type III procollagen (PCIII), type IV collagen (Col-IV), connective tissue growth factor (CTGF), and transforming growth factor-β1 (TGF-β1) and (6) improvement in TCM symptoms scores after treatment.

The primary evaluation index was the change in serum creatinine (Scr) after treatment. Renal fibrosis is the main pathological basis for the progression of CKD, and dyslipidemia, inflammation, immune responses may lead to the exacerbation of renal fibrosis. Therefore, secondary evaluation indices include changes in BUN, UC, eGFR, 24 h U-pro, etc.; serum lipid indicators (TG, TC, HDL-C, and LDL-C), inflammatory indicators (IL-1β, IL-6, and TNF-α), oxidative damage indicators (HO-1, SOD, MDA), renal fibrosis indicators (PCIII, Col-IV, CTGF, and TGF-β1), and TCM symptom scores.

#### 2.5.2 TCM syndrome score

(1) Clinical symptoms: clinical observation tables will be established to assess syndrome scores. According to “*Diagnosis, Dialectical Classification and Efficacy Evaluation of Chronic Renal Failure*” (45), the syndrome differentiation criteria were formulated as follows: main symptoms: fatigue, weak waist and knees, edema, stomach flatulence. (2) Second disease: clearer and longer urine in night, fear of cold and warm, thinner stool. Tongue: Lilac. Pulse: fine or dull. Concurrent syndrome of blood stasis: Symptoms: malformed skin, dark purple lip nails, fixed or tingling back pain. Tongue: dark purple, tongue with petechiae or spots. Pulse: astringent or fine astringent pulses. The tongue-coating pulse can only be recorded separately in the record book and will not participate in the final score addition and comparison. The scoring grades were generally 0, 1, 2, 3 points for normal, mild, moderate, and severe, respectively.

### 2.6 Evaluation criteria for TCM syndromes effect

According to the *Guidelines for Clinical Research of Chinese Medicine (New Drug)*, the symptoms of spleen-kidney qi deficiency and blood stasis syndrome were categorized as normal, mild, moderate, and severe with 0, 1, 2, 3 points, respectively. The total scores before and after treatment for each patient will be summed according to TCM symptoms, and then efficacy indicator (EI) will be calculated:

EI = decreased symptom score before and after treatment/total symptom score before treatment. EI was adopted to evaluate treatment efficacy after treatment: significantly effective (EI ≥ 60%), effective (60% > EI ≥ 30%), stable (30% > EI), and to ineffective (TCM syndrome scores did not decrease or worsen). Two professional TCM doctors will conduct TCM symptoms and syndrome assessment and scoring, and then take the average to ensure a reduction in potential bias caused by the lack of blinding and occasional factors.

### 2.7 Data entry and management

Each participant was required to complete the CRFs accurately and truthfully. It was the supervisor’s responsibility to review completed CRFs and submit them to the designated data administrators for entry and management. Data administrators used the EpiData software to perform these tasks. Personal information was kept strictly confidential, with access granted with only the supervisor’s permission. In compliance with the GCP requirements, all data were retained for more than 5 years after the completion of the trial.

### 2.8 Sample size estimation

The sample size was determined based on the primary outcome of the change in the baseline Scr. According to our previous study (46), the mean Scr value was 121.8 μmoL/L, with a standard deviation of 29.7 μmoL/L. It was hypothesized that the Scr level in the GTF + LP group would decrease significantly by more than 20 μmoL/L. Using the PASS (version 15.0.3) software, the sample size was calculated to achieve a test power of 1–β = 0.90 and a significance level of α = 0.05 (two-sided test). Considering a 20% dropout rate, the estimated sample size was 60 participants per group, for a total of 120 participants.

### 2.9 Quality control of data

Before initiating the trial, a meeting was held to ensure that all the participants received the necessary training. They underwent professional training to ensure that the CRFs was completed accurately and thoroughly. The clinical trial was designed according to GCP requirements, and participants were enrolled strictly according to established standards. The entire process followed rigorous clinical design protocols. At each site, the test samples were sent to the central laboratory for analysis and disposal to minimize data deviations. Professional biologists analyzed the collected data. Data administrators primarily conducted manual checks of data entry with systematic reviews performed upon completion. The Data Monitoring Committee regularly monitored and evaluated the quality of the data.

### 2.10 Safety assessment

Data were collected on general condition, routine hematuria, liver and renal function, electrocardiograms, and the incidence of AEs. AEs were recorded and reported, and it was essential to accurately assess the cause and effect of AEs and any drugs involved based on the relevant causal judgment indicators. If AEs occurred, the attending physician decided whether to suspend the trial based on the participant’s condition. Adverse reactions were followed up and documented in detail. In the event of serious AEs (SAEs), the investigator was required to take appropriate protective measures for the participants and report the event to the drug administrator within 24 h.

### 2.11 Statistical analysis

For all data analyses, SPSS (version 29.0) and GraphPad Prism 9 (GraphPad Software, Boston, MA, USA) were utilized. The statistics will encompass the actual recruitment numbers for the two groups, dropout and exclusion instances, demographic information, efficacy and safety metrics, and subgroup analyses according to the disease being addressed. Analysis of efficacy will be carried out using the FAS and per protocol set (PPS) datasets. At each follow-up, the main and secondary efficacy indicators were compared between the two groups, and the 95% confidence interval for the difference in symptom scores was determined. The safety dataset (SS) was used to conduct a safety analysis. Data are presented as the mean ± standard deviation and were processed using SPSS software. Continuous data were analyzed using the *t*-test or Wilcoxon rank-sum method, and categorical data were assessed using the *χ*^2^ test. To compare the frequency of adverse events and those linked to the study drug between the two groups, Fisher’s exact probability method was utilized. Descriptive statistics for lab test results will emphasize abnormal values, and cross-tabulation will be employed to illustrate changes in lab test indicators before and after treatment. For each visit, vital signs will be detailed using the mean ± standard deviation, along with the maximum and minimum values. To compare groups, a paired *t*-test or Wilcoxon rank sum test will be used for differences within groups, and a grouped *t*-test will be applied for differences between groups. A *p*-value < 0.05 was considered to indicate a statistically significant difference, while a *p-*value > 0.05 suggested no significant statistical difference between the comparative data.

## 3 Results

### 3.1 Baseline characteristics of enrolled participants

After screening 168 patients, 120 were enrolled. Of the 168 patients, 21 did not meet the inclusion criteria, 16 met the exclusion criteria, seven declined to participate, and four were excluded for other reasons (Figure 1). The LP group consisted of 32 male participants, with a mean age of 60.5 ± 9.8 years. In the LP + GTF group, there were 35 male participants, with a mean age of 56.6 ± 13.8 years. The distribution of CKD categories is shown in the table below, with diabetic nephropathy being the most prevalent, accounting for 65.0%. Statistical analysis revealed no significant differences in sex, age, or CKD categories between the two groups (*p* > 0.05), indicating that the baseline characteristics were well-balanced and the efficacy of the clinical study was comparable (Table 1).

**Table 1:** Baseline characteristics of enrolled participants.

Characteristics	Total (*n* = 120)	LP group (*n* = 60)	LP+GTF group (*n* = 60)
Male	67 (55.8%)	32 (53.3%)	35 (58.3%)
Age (years)	58.6 ± 12.1	60.5 ± 9.8	56.6 ± 13.8
Categories of CKD			
Diabetic nephropathy	78 (65.0%)	36 (60.0%)	42 (70.0%)
Hypertensive nephropathy	64 (53.3%)	29 (48.3%)	35 (58.3%)
Chronic nephritis	24 (20.0%)	14 (23.3%)	10 (16.7%)
Hyperuricemic nephropathy	43 (35.8%)	22 (36.7%)	21 (35.0%)

LP, losartan potassium; GTF, Guben Tongluo formula; CKD, chronic kidney disease.

### 3.2 Evaluation of primary outcome

**Figure 2:**
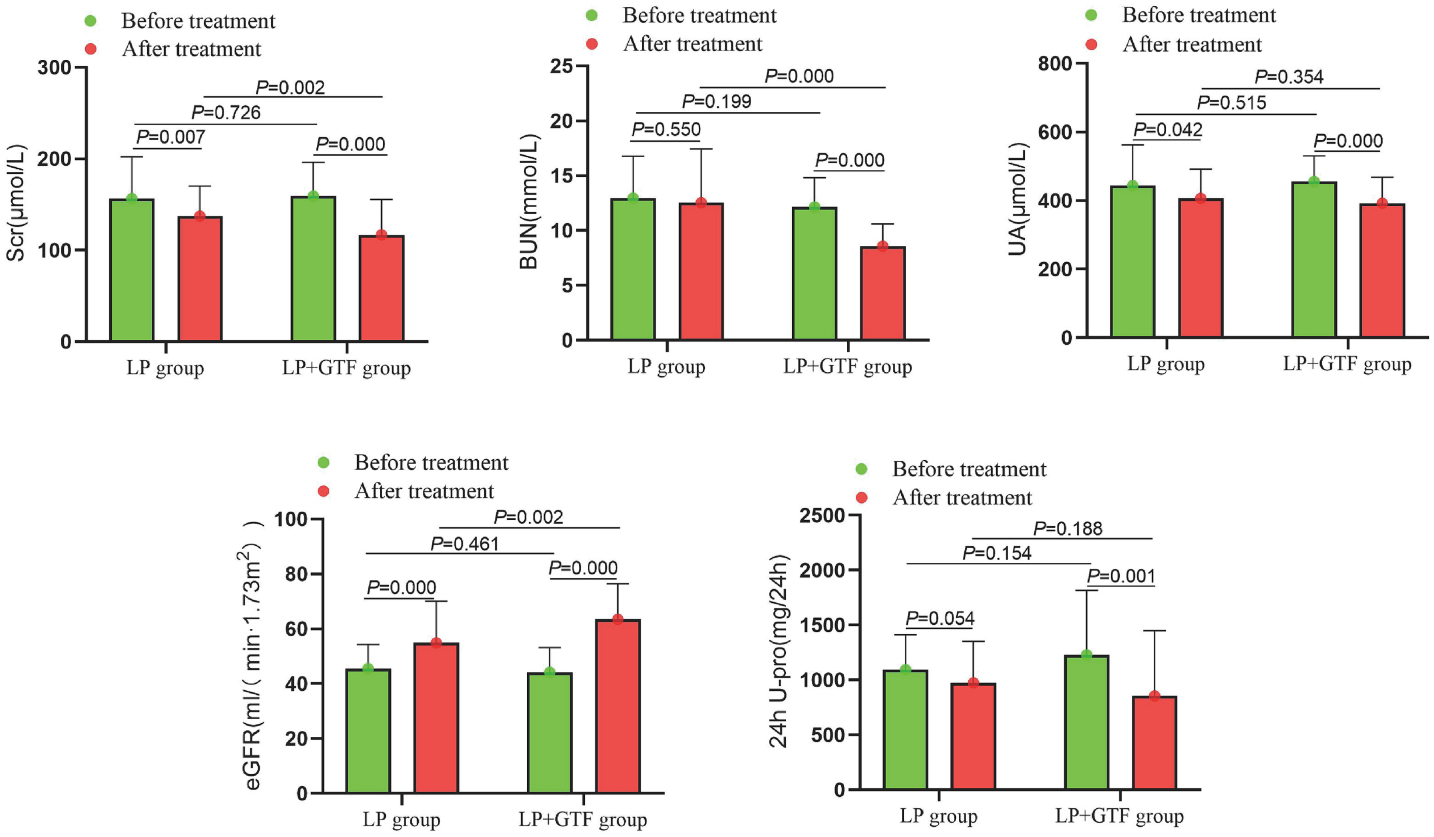
The comparison of renal function indicators between two groups before and after treatment.

The primary evaluation endpoint was the change in Scr level after treatment. Statistical analysis showed that the mean ± SD of Scr after treatment was 137.3 ± 32.8 in the LP group and 116.8 ± 38.5 in the LP + GTF group. Both groups showed a significant reduction in Scr after treatment compared to baseline (*p* < 0.01). Scr levels were significantly lower in the LP + GTF group than in the LP group (*p* < 0.05) (Table 2 and Figure 2).

**Table 2:** The comparison of primary and secondary efficacy endpoints.

Variables	LP group (*n* = 60)		LP+GTF group (*n* = 60)	
	Before treatment	After treatment	Before treatment	After treatment
Renal function indicators				
Scr (μmol/L)	156.4 ± 45.9	137.3 ± 32.8^*^	159.1 ± 37.0	116.8 ± 38.5^*#^
BUN (mmol/L)	12.9 ± 3.8	12.5 ± 4.9	12.1 ± 2.7	8.6 ± 2.0^*#^
UA (μmol/L)	443.7 ± 118.7	406.6 ± 85.1^*^	455.3 ± 74.8	392.4 ± 76.1^*^
eGFR [mL/(min·1.73m^2^)]	45.4 ± 8.8	54.9 ± 15.1^*^	44.1 ± 8.9	63.5 ± 12.9^*#^
24h U-pro (mg/24h)	1092.1 ± 319.5	974.3 ± 377.7	1227.3 ± 590.5	854.7 ± 594.8^*^
Serum lipid indicators				
TG (mmol/L)	2.1 ± 1.0	1.6 ± 0.6^*^	2.3 ± 1.2	1.3 ± 0.4^*#^
TC (mmol/L)	4.9 ± 1.4	4.4 ± 1.1^*^	5.2 ± 1.6	3.3 ± 1.0^*#^
HDL-C (mmol/L)	1.2 ± 0.4	1.3 ± 0.5^*^	1.6 ± 0.6	2.0 ± 0.9^*#^
LDL-C (mmol/L)	3.5 ± 1.6	4.0 ± 1.0^*^	3.6 ± 2.0	3.8 ± 0.9
Inflammatory factors				
IL-1β (pg/mL)	13.7 ± 3.4	11.6 ± 3.3^*^	13.9 ± 3.0	10.3 ± 1.8^*#^
IL-6 (pg/mL)	14.1 ± 3.4	13.7 ± 3.4^*^	12.9 ± 2.5	10.8 ± 2.2^*#^
TNF-α (pg/mL)	37.8 ± 6.3	35.1 ± 8.1^*^	38.7 ± 8.4	19.9 ± 6.8^*#^
Oxidative damage indicators				
HO-1 (ng/mL)	78.0 ± 6.3	77.6 ± 8.4	77.9 ± 7.5	91.4 ± 14.0^*#^
SOD (U/mL)	65.5 ± 7.9	68.1 ± 6.6	67.6 ± 8.0	87.3 ± 7.3^*#^
MDA (nmol/mL)	13.1 ± 3.6	12.1 ± 3.6	13.7 ± 2.7	11.6 ± 5.9^*^
Renal fibrosis indicators				
PC-III(ng/mL)	115.0 ± 24.0	91.4 ± 16.0^*^	117.9 ± 20.5	87.1 ± 13.2^*^
Col-IV(ng/mL)	151.3 ± 28.4	91.8 ± 13.9^*^	149.1 ± 26.8	87.1 ± 10.6^*^
CTGF (ng/mL)	108.1 ± 11.8	102.4 ± 24.2	112.5 ± 12.0	90.5 ± 11.9^*#^
TGF-β_1_ (ng/mL)	103.4 ± 11.2	107.6 ± 10.5	106.9 ± 10.1	92.5 ± 13.5^*#^
TCM syndrome scores	57.2 ± 11.5	45.6 ± 14.9^*^	59.5 ± 11.5	33.3 ± 11.2^*#^

LP, losartan potassium; GTF, Guben Tongluo formula; Scr, serum creatinine; BUN, blood urea nitrogen; UA, uric acid; eGFR, estimated glomerular filtration rate; 24 h U-pro, 24-h urine protein; TG, triglyceride; TC, cholesterol; HDL-C, high-density lipoprotein cholesterol; LDL-C, low-density lipoprotein cholesterol; IL-1β, interleukin-1β; IL-6, interleukin-6; TNF-α, tumor necrosis factor-α; HO-1, Hemeoxygenase-1; SOD, superoxide dismutase; MDA, malonaldehyde; PC-III, type III procollagen; Col-IV, type IV collagen; CTGF, connective tissue growth factor; TGF-β_1_, transforming growth factor-β_1_; TCM, traditional Chinese medicine. *Statistically significant difference from before treatment. ^#^Statistically significant difference from LP group; *p* < 0.05 was considered statistically significant difference.

### 3.3 Evaluation of secondary outcomes

#### 3.3.1 Comparison of other renal function indicators

Other indicators of renal function were assessed. The mean ± SD of BUN after treatment was 12.5 ± 4.9 in the LP group and 8.6 ± 2.0 in the LP + GTF group. BUN levels were significantly lower in the LP + GTF group than in the LP group (*p* < 0.01). UA and 24 h U-pro levels were lower in the LP + GTF group after treatment than at baseline, but there was no statistically significant difference compared to the LP group (*p* > 0.05). eGFR improved in the LP + GTF group, with a statistically significant difference (*p* < 0.01) (Table 2 and Figure 2).

#### 3.3.2 Comparison of serum lipid indicators

**Figure 3:**
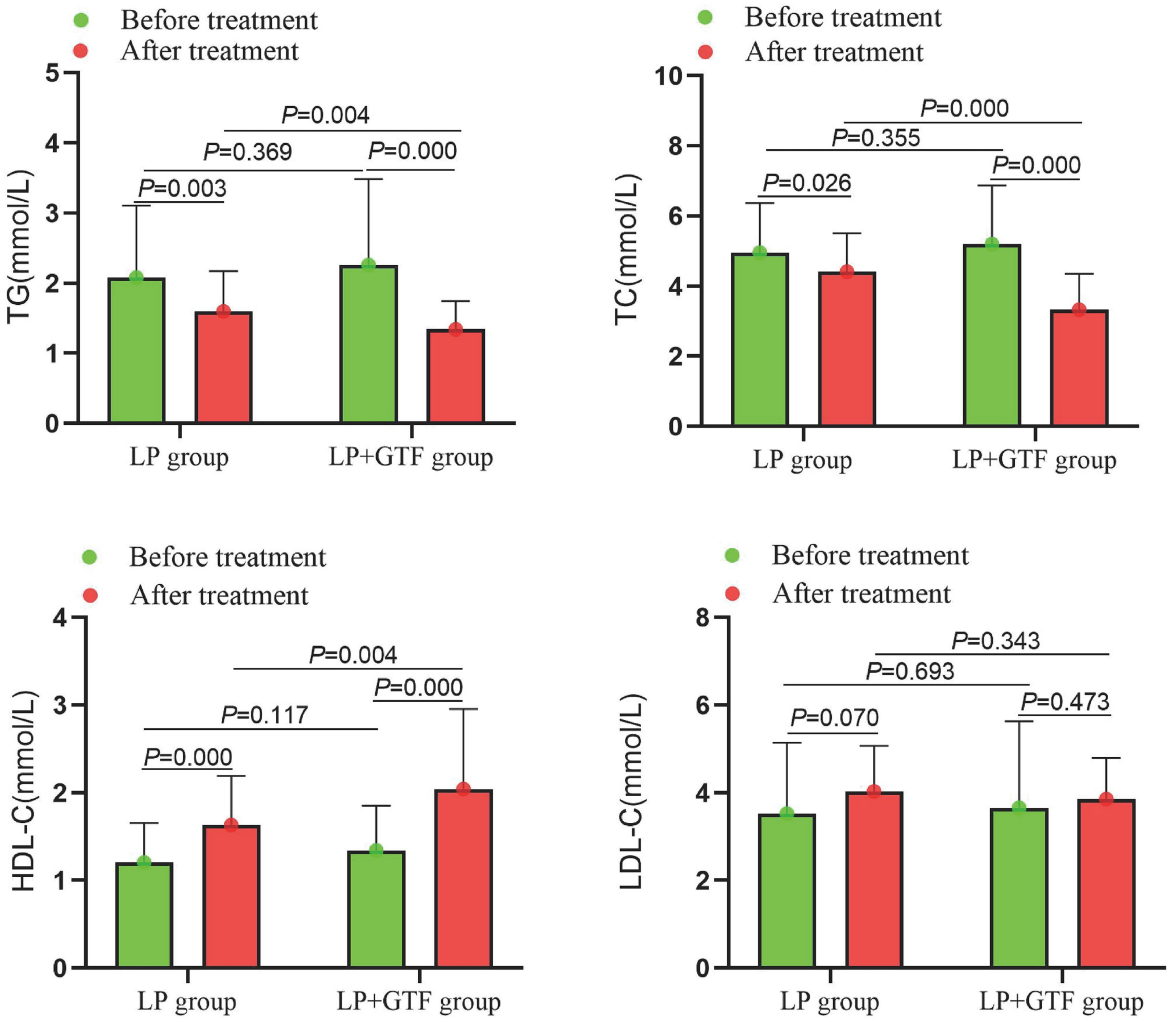
The comparison of serum lipids indicators between two groups before and after treatment.

For lipid profiles, the mean ± SD of TG after treatment was 1.6 ± 0.6 in the LP group and 1.3 ± 0.4 in the LP + GTF group. The TG levels were significantly lower in the LP + GTF group than in the LP group (*p* < 0.01). Additionally, TC levels were significantly lower and HDL-C levels were significantly higher in the LP + GTF group than in the LP group (*p* < 0.01). No statistically significant difference in LDL-C levels was observed between the two groups (Table 2 and Figure 3).

#### 3.3.3 Comparison of inflammatory factors and oxidative damage indicators

**Figure 4:**
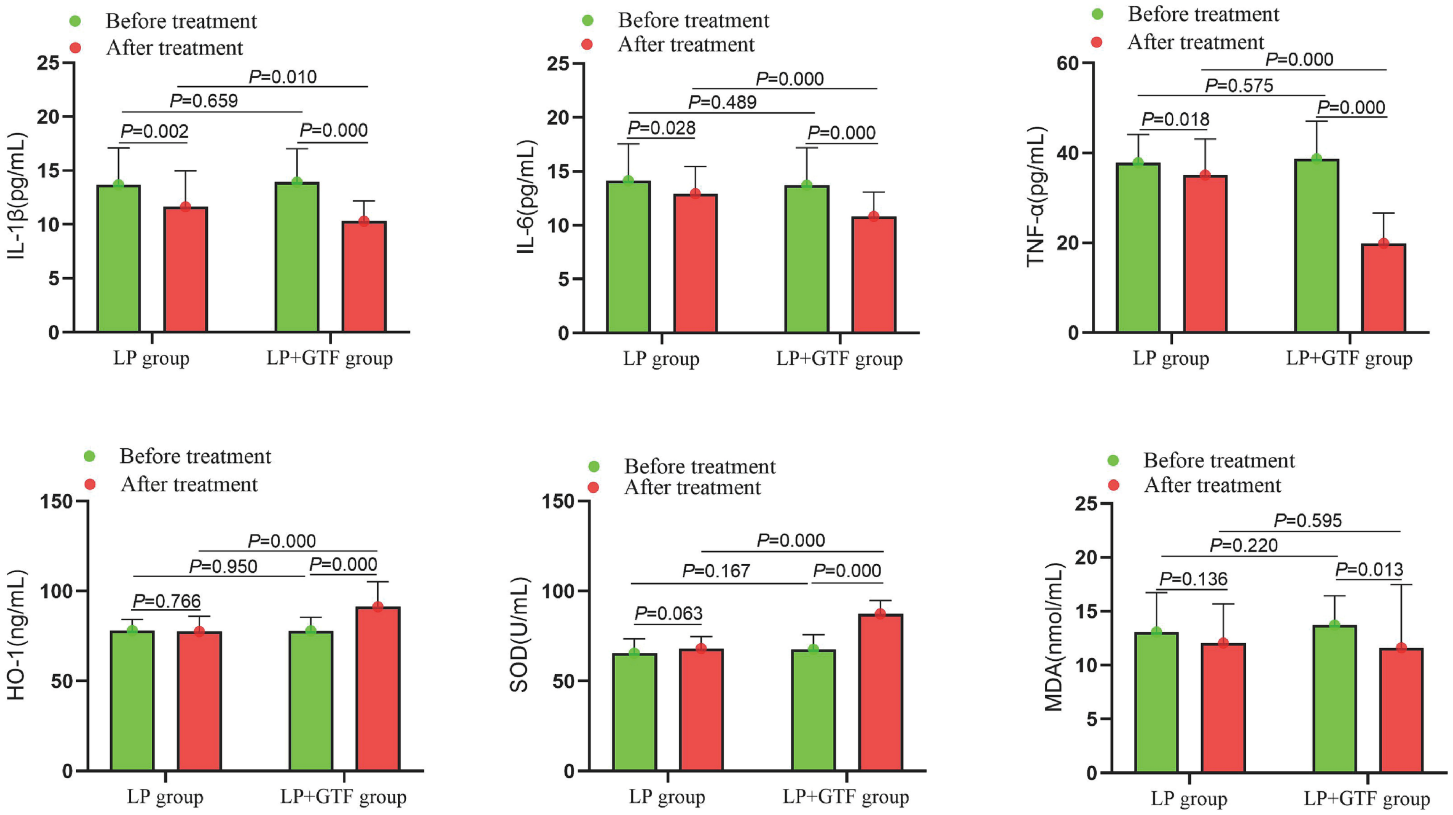
The comparison of inflammatory factors and oxidative damage indicators between two groups before and after treatment.

Statistical analysis revealed that the mean ± SD of IL-1β after treatment was 11.6 ± 3.3 in the LP group and 10.3 ± 1.8 in the LP + GTF group. IL-1β levels were significantly lower in the LP + GTF group than in the LP group (*p* < 0.01). Additionally, IL-6 and TNF-α levels were significantly lower in the LP + GTF group (*p* < 0.01). Notable increases in HO-1 and SOD levels were observed in the LP + GTF group, with a significant difference (*p* < 0.01). MDA levels showed more pronounced changes in the LP + GTF group after treatment (*p* < 0.05), but no statistically significant difference was found compared with the LP group (*p* > 0.05) (Table 2 and Figure 4).

#### 3.3.4 Comparison of renal fibrosis indicators

**Figure 5:**
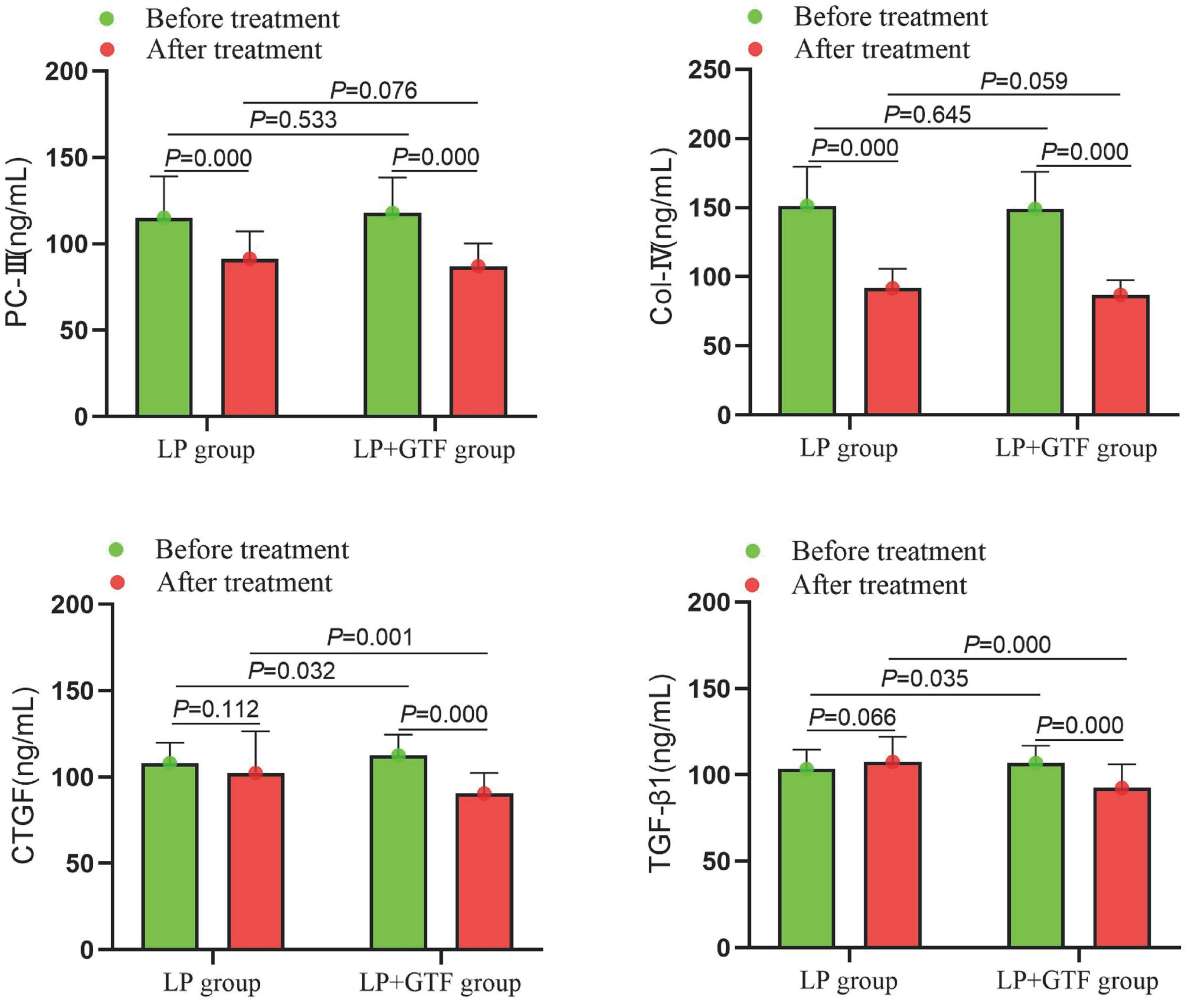
The comparison of renal fibrosis indicators between two groups before and after treatment.

The mean ± SD of CTGF after treatment was 102.4 ± 24.2 in the LP group and 90.5 ± 11.9 in the LP + GTF group. CTGF levels were significantly lower in the LP + GTF group than in the LP group (*p* < 0.01). Similarly, TGF-β1 levels were significantly lower in the LP + GTF group (*p* < 0.01). PC-III and Col-IV levels were reduced in the LP + GTF group after treatment (*p* < 0.01), although no significant difference was observed compared with the LP group (*p* > 0.05) (Table 2 and Figure 5).

#### 3.3.5 Comparison of TCM syndrome scores

**Figure 6:**
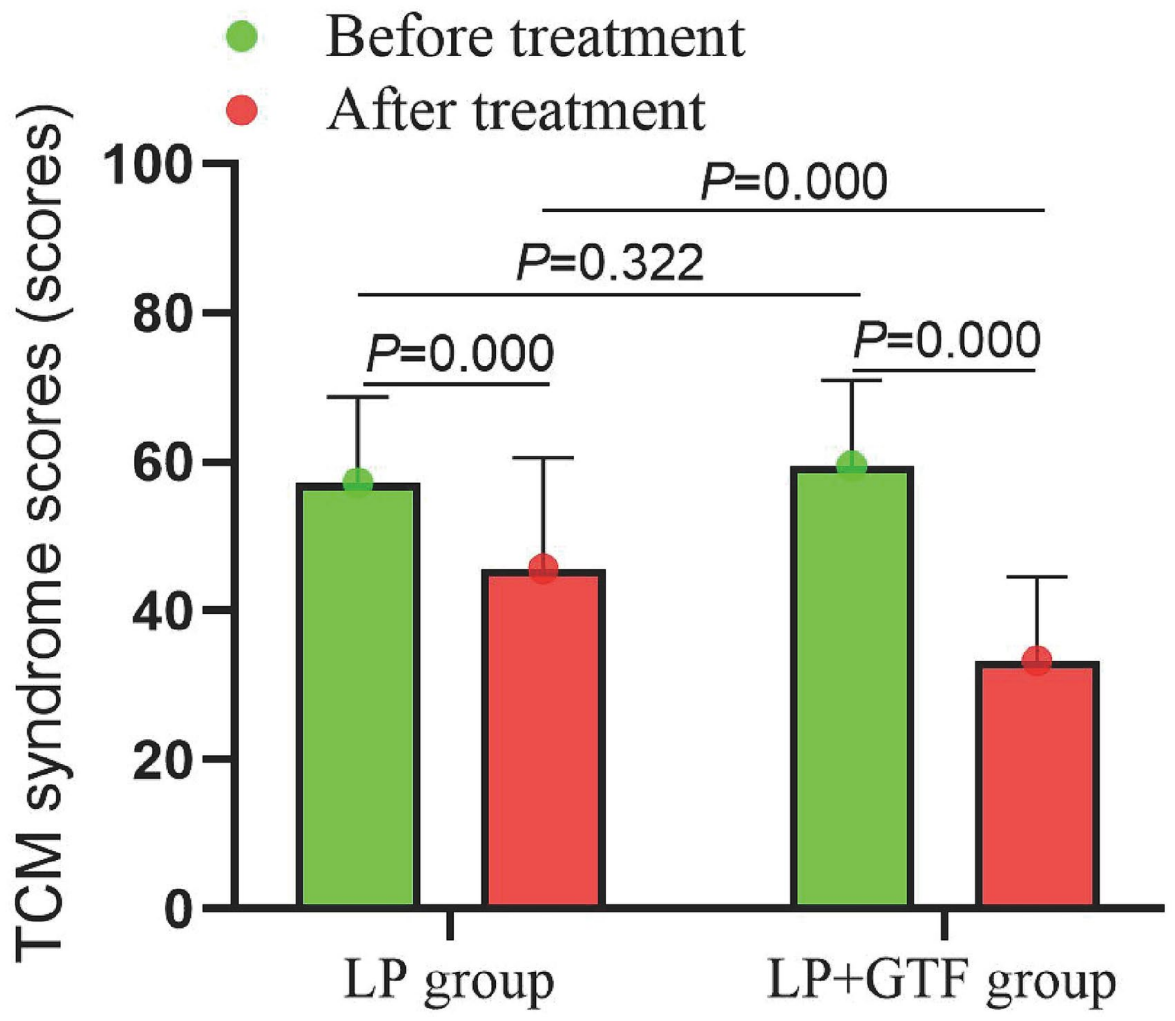
The comparison of TCM syndrome scores between two groups before and after treatment.

Statistical results showed that mean ± SD of TCM syndrome scores, respectively, were 45.6 ± 14.9, 33.3 ± 11.2 in the LP group and LP + GTF group after treatment. Compared with the LP group, TCM syndrome scores in the LP + GTF group were significantly lower (*p* < 0.01) (Table 2 and Figure 6).

#### 3.3.6 Comparison of clinical effective rate

As presented in Table 3, the significant effective and effective rates in the LP group were 7 (11.7%) and 17 (28.3%), respectively. In contrast, the significant effective and effective rates in the LP + GTF group were 18 (30%) and 26 (43.3%), respectively. The total efficacy rate in the LP + GTF group (73.3%) was significantly higher than that in the LP group (40%) (Table 3).

**Table 3:** The comparison of clinical effective rate between two groups.

Effective rate	LP group (*n* = 60)	LP + GTF group (*n* = 60)
Significantly effective	7 (11.7%)	18 (30%)
Effective	17 (28.3%)	26 (43.3%)
Stable	19 (31.7%)	12 (20%)
Ineffective	17 (28.3%)	4 (6.7%)
Total effective rate	24 (40%)	44 (73.3%)

LP, losartan potassium.

#### 3.3.7 Safety evaluation

The AEs that occurred in the LP and LP + GTF groups are listed in Table 4. No SAEs requiring withdrawal were reported during the treatment period in either group. In the LP group, two participants experienced AEs (gastrointestinal reactions and skin rash), while in the LP + GTF group, three participants experienced AEs (gastrointestinal reactions, dizziness, or headache).

**Table 4:** Adverse events during treatment period.

Adverse events	LP group (*n* = 60)	LP + GTF group (*n* = 60)
Overall	2	3
Severe adverse events	0	0
Gastrointestinal reactions	1	2
Dizziness or headache	0	1
Arrhythmia	0	0
Skin rash	1	0
Other adverse events	0	0

LP, losartan potassium.

## 4 Discussion

At stage G3 of CKD, most patients begin to exhibit clinical symptoms, indicating that the kidney’s compensatory capacity can no longer meet the body’s normal demands (47–49). Effective and standardized treatments may still offer the potential for clinical improvement (50–53). However, without proper control and monitoring, the condition often progresses rapidly to ESRD, accompanied by anemia, dizziness, fatigue, nausea, and other irreversible complications (54–56). With growing health awareness, the focus of CKD prevention and treatment has shifted from managing ESRD to intervening in the early stages of CKD. Thus, identifying a safe, effective, simple, and affordable treatment strategy to delay progression to the final stage is not only beneficial for the majority of patients with CKD but also represents a significant milestone in nephrology with considerable practical and societal value (57–59).

In patients with CKD, toxins can induce systemic inflammation, immune dysfunction, oxidative stress, and elevated risk of vascular disease (60–63). These complications are strongly associated with low survival rates observed in patients with CKD (64). CKD is often accompanied by immune dysfunction, primarily involving cellular immune impairment (65). Immune system disturbances are common in patients with CKD. During CKD progression, numerous inflammatory mediators, such as IL-6, IL-1β, and TNF-α, are released. These pro-inflammatory cytokines drive inflammatory infiltration in the renal interstitium and trigger the release of nephrotoxic factors from kidney resident cells, leading to the continuous proliferation of fibroblasts and eventual development of renal fibrosis (66–68). CKD is characterized by the dual presence of oxidative stress and inflammation, both of which contribute to disease progression and the onset of cardiovascular complications (69–71). As CKD advances, abnormal extracellular matrix deposition in the renal tubules leads to renal fibrosis, ultimately resulting in ESRD (72–74).

TCM has been utilized in the treatment of acute and chronic kidney diseases for thousands of years and has demonstrated remarkable clinical efficacy (75–77). Natural products are recognized as sources of new drugs over the nearly four decades (78–81). In the GTF formulation, Raw *Astragalus* was employed to strengthen spleen qi and alleviate edema. *Salvia Miltiorrhiza*, *Peach Kernel*, *Herba Lycopi*, and *Ramuli Euonymi* promote blood circulation, resolve stasis, expel turbid substances, and support diuresis (82–85). *Glossy Privet* Fruits and *Eclipta Alba* primarily tonify the kidneys and strengthen their essence. *Rheum Officinale* and *Rhizoma Imperatae* eliminate accumulations, clear heat, and cool the blood (78, 86). The complete formula harmonizes the functions of tonifying the spleen and kidneys, resolving blood stasis, and expelling turbidity.

Currently, it is believed that LP can effectively lower blood pressure, reduce the risk of cardiovascular diseases, and alleviate renal damage (87). But it can cause arrhythmia, increase the burden on the gastrointestinal tract, lead to headaches and dizziness, as well as cause kidney damage. When treating CKD, angiotensin-converting enzyme inhibitors (ACEIs)/angiotensin receptor blockers (ARBs) and sodium-glucose co-transporter-2 (SGLT-2) inhibitors are two commonly used drugs (88). The adoption of ACEI/ARB will be beneficial for reduction of proteinuria and blood pressure control, while it may lead to deterioration of renal function. SGLT2 inhibitors may have cardiorenal protective effect and delay the prognosis of CKD (89). But the application of SGLT2 inhibitors might increase risk of urinary tract infection and cause dehydration. As demonstrated in the study, GTF showed significant reno-protective effects and has relatively minor side effects, which might be an alternative drug method for CKD treatment.

Furthermore, GTF enhances renal function in patients with chronic glomerulonephritis. As a key component of the inflammatory response, nod-like receptor pyrin domain-containing 3 (NLRP3) is closely associated with immune inflammation (90). Ongoing inflammation is a key factor in the development and issues associated with CKD. The NLRP3 inflammasome pathway has become an essential player in mediating the inflammatory response in CKD (91). Increased levels of NLRP3 in hemodialysis patients imply a potential link between the inflammasome and oxidative stress in CKD patients (92). By blocking ferroptosis, the accumulation of profibrotic cytokines is effectively reduced, which helps in relieving renal fibrosis. The TGF-β/Smad pathway is intricately linked to the profibrotic mechanism of ferroptosis, and enhancing GPX4 expression while targeting ferroptosis might be a promising approach for CKD treatment (93–95). GTF effectively improves albuminuria in IgA nephropathy mice, and its mechanism is potentially linked to the regulation of NLRP3 expression (40). LP has been shown to significantly reduce the combined risk of cardiovascular diseases. Based on prior clinical experience, GTF has proven to be effective in alleviating renal injury and reducing proteinuria. As this clinical trial progresses, it is expected to offer deeper insights into the mechanisms through which GTF alleviates CKD, particularly through the anti-inflammatory, antioxidant, and anti-renal fibrosis pathways. Moreover, the combination of GTF and LP is expected to provide a novel therapeutic approach for the clinical management of CKD. The findings of this study will offer a scientific framework for future clinical trials, lay the groundwork for further basic research, and open avenues for exploring the multi-target and multi-pathway potential of TCM in the prevention and treatment of CKD.

This study had several limitations. Despite meeting statistical requirements, the study remains a relatively small-scale trial with limited follow-up, which may restrict generalizability. In addition, owing to the constraints of the dosage form, a complete blinding method could not be implemented, which may have influenced the results. Furthermore, variations in participants’ underlying conditions could introduce uncertainty into trial outcomes. Future studies should expand the sample size and optimize trial procedures to minimize the impact of research biases on the results, as well as address more comprehensively potential confounders (concomitant medications, missing data and multiplicity).

## 5 Conclusion

The LP + GTF group exhibited a higher clinical efficacy than the LP group. GTF demonstrated significant reno-protective effects, regulated serum lipid levels, alleviated clinical symptoms, and delayed the progression of renal fibrosis. The potential mechanisms underlying these effects may involve anti-inflammatory, antioxidative, and anti-renal fibrotic properties. GTF showed potential benefits in this small multicenter randomized controlled trial, warranting confirmation in larger, fully blinded randomized trials.

## Data Availability

The original contributions presented in the study are included in the article/supplementary material, further inquiries can be directed to the corresponding authors.
